# Clinical features and epidemiological analysis of respiratory human adenovirus infection in hospitalized children: a cross-sectional study in Zhejiang

**DOI:** 10.1186/s12985-021-01705-x

**Published:** 2021-11-29

**Authors:** Caiyun Wang, Juanjuan Liu, Yumei Mi, Jing Chen, Jing Bi, Yinghu Chen

**Affiliations:** 1 Department of Infectious Disease, Children’s Hospital of Zhejiang University School of Medicine, National Clinical Research Center for Child Health, National Children’s Regional Medical Center, 3333 Binsheng Road, Hangzhou, 310052 China; 2 Department of Otolaryngology-Head and Neck Surgery, Children’s Hospital of Zhejiang University School of Medicine, National Clinical Research Center for Child Health, National Children’s Regional Medical Center, 3333 Binsheng Road, Hangzhou, 310052 China

**Keywords:** Human adenovirus, Acute respiratory infections, Molecular epidemiology, Children

## Abstract

**Background:**

HAdV is one of the common pathogens in hospitalized children with acute respiratory infections (ARIs). We aim to describe the clinical and laboratory features, epidemiological characteristics, and HAdV species and/or types of inpatients with HAdV respiratory infections.

**Methods:**

Respiratory samples were gathered from inpatients diagnosed ARIs in Children’s Hospital, Zhejiang University School of Medicine, and were detected by using Direct Immunofluorescence Assay from 2018 to 2019. PCR amplification and sequencing of the hypervariable zone of *hexon* gene were used for genotyping. The clinical and laboratory features, and HAdV genotyping, and epidemiological characteristic analysis were retrospectively performed.

**Results:**

Of 7072 samples collected, 488 were identified as HAdV-positive. The overall detection rate was 6.9%. The peaked detection rate was 14.1% in January 2019. HAdV-positive cases with ARIs mainly appeared in winter. The detection rate was highest among children between 6 months and 2 years (8.7%, 123/1408). Clinical diagnosis included pneumonia (70.3%, 343/488), bronchitis (7.0%, 34/488) and acute upper respiratory tract infection (22.7%, 111/488). The common clinical manifestations were fever (93.4%, 456/488), cough (94.7%, 462/488), wheezing (26.2%, 128/488), and shortness of breath (14.8%, 72/488). 213 (43.6%) cases had co-infection and 138 (28.3%) cases had extrapulmonary symptoms. 96(19.7%) cases had intrapulmonary and intrathoracic complications.78 (16.0%) had an underlying condition, most of which were congenital heart diseases (20.5%, 16/78). The proportions of hyperpyrexia, duration of fever > 10 days, severe pneumonia, and wheezing in the co-infection group were remarkably higher than those in HAdV single-infection group (all *p* < 0.05). The proportions of duration of hospitalization, duration of fever > 10 days, wheezing, shortness of breath, change in level of consciousness, serosal fluids, extrapulmonary symptoms, co-infections and underlying diseases were significantly higher in severe pneumonia group than those in the mild pneumonia group (all *p* < 0.05). Four HAdV species were successfully identified in 155 cases and presented by 8 genotypes. HAdV-B3 (56.1%, 87/155) and HAdV -B7 (31.0%, 48/155) were the most predominant detected types and occurred commonly in different severity groups (*p* = 0.000), while, HAdV-B55 was detected only in the severe group. HAdV-B7’s detection rate in the severe pneumonia group was significantly higher than the non-severe pneumonia group.

**Conclusion:**

HAdV detection rate is related to age and season. Bronchopneumonia accounts for about 70% HAdV-positive inpatients. The common clinical manifestations include hyperpyrexia, cough, wheezing, and shortness of breath. HAdV-B3 and HAdV-B7 are the most common types in children diagnosed with respiration infections.

## Introduction

Human adenovirus (HAdV) is one of the most common pathogens of acute respiratory infections in children, which account for approximately 5–10% of all acute respiratory illnesses (ARIs) in children [1–4]. Although HAdV infections cause light to moderate ARIs within immunocompetent children, a serious condition may occur in younger children [5], immunocompromised patients, and those with underlying chronic diseases which can cause significant morbidity and mortality [6, 7]. HAdV often causes respiratory adenovirus epidemics and outbreaks in different provinces of China [8, 9]. Re-emergence of HAdV epidemic in central and southern China in late 2018 and 2019 [10, 11]. HAdV genotypes causing localized outbreaks or epidemics of respiratory infections in specific regions may be different or new types may emerge, presenting a complex and variable molecular epidemiological profile [12].

It is necessary to strengthen surveillance of HAdV and observe its epidemic and infection characteristics. In this study, we retrospectively analyzed the data on clinical and laboratory features, HAdV types and/or species, and epidemiological characteristics in hospitalized children with acute respiratory infection in Children’s Hospital of Zhejiang University School of Medicine from August 2018 to September 2019 to provide a theoretical basis for early and rapid clinical recognition of severe HAdV infections.

## Materials and methods

### Study design, case definition, and identification

From August 2018 to September 2019, samples of the nasopharyngeal aspirates (NPAs) or nasal/nasopharyngeal swabs (in viral transport media) were collected from hospitalized children aged  < 18 years who presented with ARIs at Children’s Hospital of Zhejiang University School of Medicine, Zhejiang Province, China. We performed respiratory virus identification by using D3 *Ultra*^TM^ DFA Respiratory Virus Screening & ID Kit (Diagnostic Hybrids, Inc., USA), which can detect *Respiratory syncytial virus* (RSV), *human adenovirus*, *influenza* A *viruses* (*Flu* A), and *influenza* B *viruses* (*Flu* B), and *parainfluenza* 1, 2, and 3 *viruses.*

All nasopharyngeal aspirates (NPAs) or nasopharyngeal swabs samples were collected within 24 hours after admission. The specimens were divided into two parts and preserved in standard transport media. Antigen testing for specimens was performed based on the instruction of the manufacturer within 2 hours after collection. All the samples were stored immediately in a tube containing 1.5 ml of viral transport medium at -80℃ for further analysis. Meanwhile, bronchial alveolar lavage fluids (BALF), pleural effusion (PE) (if available), and blood from HAdV-positive patients during hospital stay were used to identify other pathogens (Mycoplasma pneumoniae, Epstein-Barr virus, etc.) by PCR amplification.

Patient age was defined according to the date of hospitalization. All participants must satisfy the inclusion criteria as follows:

1. Aged < 18 years old and > 28 days,
2. Disease course on admission ≤ 1 week
3. Confirmed ARIs with fever at initial presentation, cough, nasal obstruction, expectoration, sneeze, dyspnea, tachypnea, dyspnea, or wheeling/rales upon auscultation.
4. HAdV positive in the testing of NPAs or nasopharyngeal swabs samples through direct immunofluorescence assay.
5. Provision of informed consent

The exclusion criteria were:

1. Acquired respiratory infection in hospital
2. Died or discharged within 48 h after admission
3. Children with immunodeficiency including neurological diseases, inherited metabolic diseases, asplenia, cerebral palsy, and/or complex- conditions.
4. Cases with substantial missing data.
5. Non-ARIs indication for hospitalization
6. Had two or more HAdV-positive respiratory specimens during the study period

### Data collection

Patient general demographics (including age, sex, and sample collection time), social, clinical characteristics, laboratory results, radiological findings, and disease severity of HAdV positive respiratory infection children admitted to our hospital were collected from the medical records after the patients’ discharge from the hospital. All the data were entered into a standard electronic database. Underlying medical diseases included congenital or acquired heart disease, asthma, epilepsy, liver, and gastrointestinal diseases, kidney disease, diabetes, or other endocrine diseases.

### Definition of clinical severity

A total of 343 patients with pneumonia were divided into a non-severe pneumonia group and a severe pneumonia group according to illness condition. Children and infants who have severe pneumonia, as defined by several factors, including respiratory distress and hypoxemia according to the American Thoracic Society’s guideline for the management of community-acquired pneumonia [13].

### Detection of HAdV and molecular genotyping

Viral nucleic acids were extracted from specimens of the HAdV-positive NPAs or swabs using QIAamp MinElute Virus Spin Kit (QIAGEN, German) based on instructions of the manufacturer. Nested-PCR amplification of coding sequences of adenovirus hexon hypervariable regions HVR1 to HVR6 was performed for determining HAdV genotyping [14]. The outer primer used for nested-PCR were forward 5′-GATGCCGCAGTGGKCKTACATG-3′ and reverse5′-GCTTACAAYTCNCTSGCT-3′, and the internal primers were forward 5′-GACGCYTCGGAGTACCTGAG-3′and reverse 5′-GGCYAGCACNTACTTTGACATYCG-3′. Nested-PCR was carried out in 25 μL volume comprising 2.5 μL of 10 × EX Taq buffer with 0.6 µL/25 mM MgCl_2_, 1.0 μL (10 pM) of each primer, 2.0 μL of dNTP Mixture, 0.5 μL of EX Taq DNA polymerase, 2.0 μL of viral nucleic acid extract or first nested-PCR product, and 16 μL of double-distilled water. The reaction conditions for both round comprised an initial denaturation step at 94 °C for 10 min, followed by 36 cycles of denaturation at 94 °C for 1 min, annealing at 55 °C for 1 min, and extension at 72 °C for 2 min, and a 7 min final extension at 72 °C. PCR reaction mixtures were analyzed on 1.0% agarose gels, purified, and were visualized under UV light with ethidium bromide containing 0.5 µg/mL(Sigma, ABD), and then confirmed as authentic by sequencing. Samples that failed to amplify are defined as untyped. Expected product size of amplifications from PCR ranged from 588 to 821 bp. Positive HAdV amplification products were sent to Beijing Genomics Institute (BGI) for Sanger sequencing of hypervariable regions of *hexon* gene. Sequenced results were compared with GenBank database of U.S. National Center for Biotecnology (NCBI).

HAdV-positive patients were divided into 3 major groups (severe pneumonia group, non-severe pneumonia group, and acute upper respiratory tract infection group) according to clinical manifestations, and patients in each group were randomly numbered. Three sets of random numbers were generated using an online random number generator in accordance with established proportions, patients were randomly sampled from each of the three groups of patients for genotype testing.

### Statistical analysis

The results were analyzed utilizing SPSS software (version 19.0). Continuous data were expressed by means and standard deviations (SDs) for normal distribution, and median (Q1, Q3) for non-normal distribution, Kruskal–Wallis *H* test was used to analyze statistical difference which *P *value was set at less than 0.05 with statistical significance. Categorical data were expressed in frequency and percentage, chi-square test and Fisher's exact test were used to analyze the statistical difference, Bonferroni chi-square segmentation was performed to compare the intergroup rates, which corrected *P *value was set at less than 0.007 with statistical significance.

## Results

### Characteristics of inpatient children and HAdV detection

**Fig. 1:**
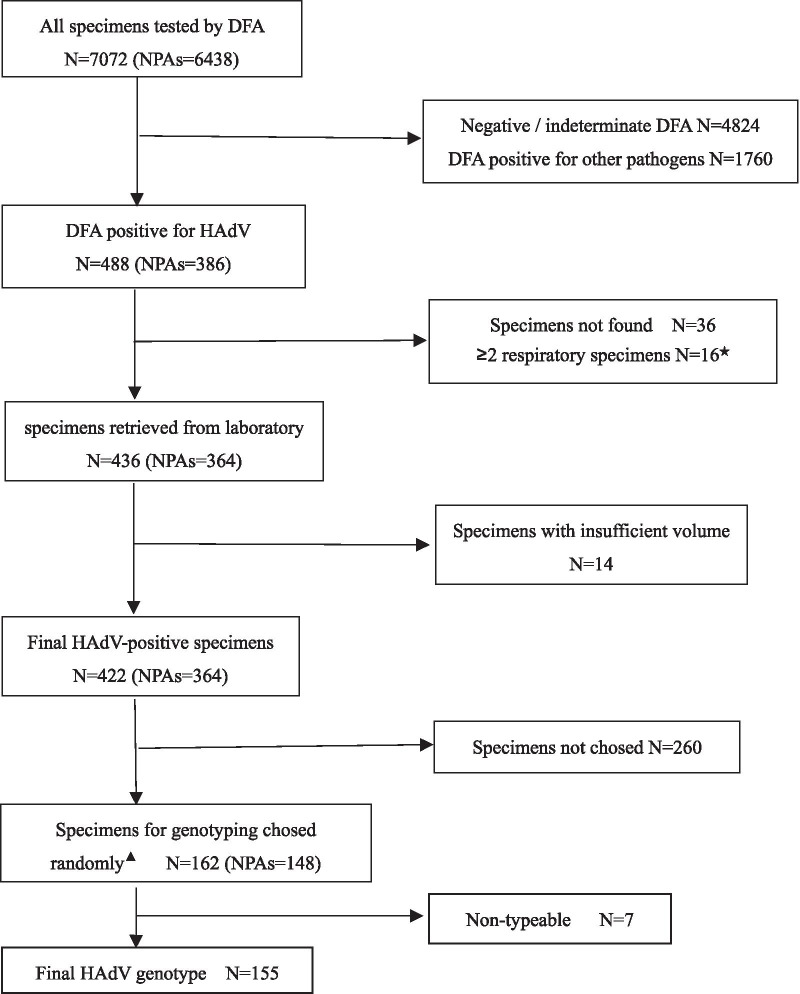
Study inclusion algorithm and result of respiratory specimens testing. Abbreviation: DFA, direct immunofluorescence assay; HAdV, human adenovirus. ^★^Two or more respiratory specimens were tested by DFA from one patients during study period. ^▲^Using an online random number generator to generate random numbers for adenovirus genotyping

In this study, a total 7072 specimens were tested by DFA from hospitalized children with ARIs in our hospital; of which, 488 were detected HAdV positive (Fig. 1). 36 specimens were missing, and 14 specimens’ volume was insufficient for genotype testing, and 16 cases had two or more HAdV-positive respiratory specimens. 162 (38.4%) specimens were eventually sampled by random number generation software for genotyping analysis (Fig. 1).

The overall HAdV detection rate was 6.90%, including 305 males (62.5%) and 183 females (37.5%), with a sex ratio of 1.68:1. The positive detection rate was 7.04% (305/4330) and 6.7% (183/2742) for male and female subjects respectively, with no significant gender difference observed (χ^2 ^= 0.358, *p *= 0.550). The median age of onset for 488 HAdV-positive children was 43 months (39 d-12 years). The age distribution of HAdV prevalence was: < 6 months (6, 1.2%), 6 months to < 2 years (123, 25.2%), 2 years to < 5 years (270, 55.3%) and ≥ 5 years (89, 18.2%). The detection rates for each group were 3.0% (6/197), 8.7% (123/1408), 7.7% (270/3519) and 4.6% (89/1948) respectively. The rate of HAdV detection was higher in the 6 months to <2 years group than in the <6 months and ≥ 5 years groups, with statistically significant differences (χ^2^=7.57, 23.98, all *P* <0.007), the rate of HAdV detection was higher in the 2 to 5 years group than in the < 6 months group and ≥ 5 years groups (χ^2^ = 5.809, 19.688, all *P* < 0.007)

**Fig. 2:**
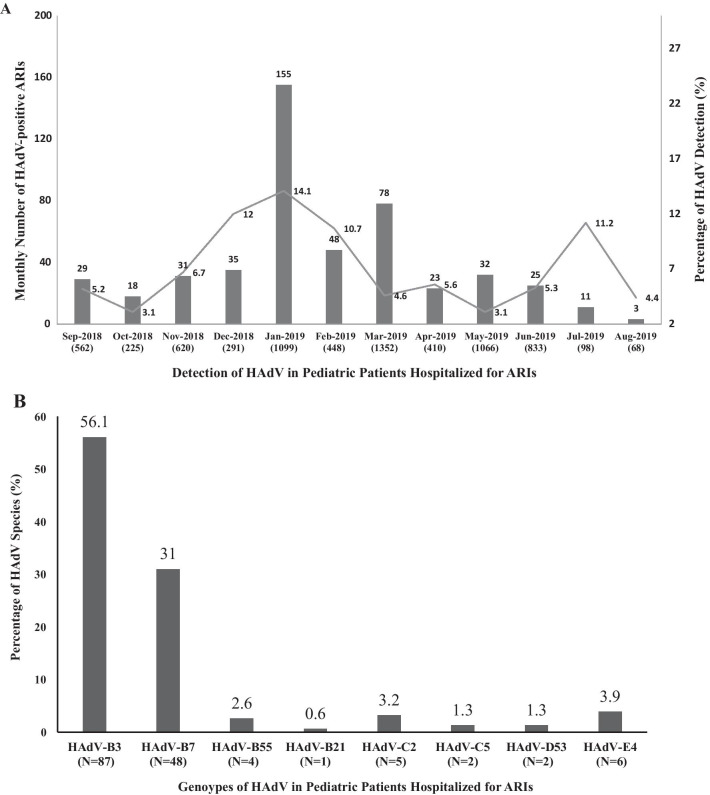
**A** Adenovirus detections by month from September 2018 to August 2019. **B** Distribution of HAdV Types among ATIs cases

The rate of HAdV detection in hospitalized children in spring, summer, autumn and winter was 4.7% (133/2,831), 3.9% (39/990), 5.5% (78/1,411) and 12.9% (238/1,840). The number of detections peaked in winter, with statistically significant differences compared to the detection rates in spring, summer, and autumn (χ^2^ = 103.477, 58.986, 49.926, all *P *< 0.007). The detection rate of HAdV in hospitalized children by month from September 2018 to August 2019 is shown in Fig. 2A, with the highest detection rate of 14.1% in January 2019.

### Clinical diagnosis and clinical characteristics of the HAdV-positive inpatients

As Table 1 shows, of the 488 HAdV-positive children, 111 (22.7%) had an upper respiratory infection, 34 (7.0%) had bronchitis and 343 (70.3%) had pneumonia, of which 86 (86/343, 25.0%) had severe pneumonia. Fever (456, 93.4%) and cough (462, 94.7%) are the most common symptoms in the HAdV-positive patients, while the other clinical presentations were wheezing (128, 26.2%), shortness of breath (72, 14.8%), and extrapulmonary symptoms (Table 1). The extrapulmonary symptoms included hepatic dysfunction, cardiac dysfunction, coagulation abnormalities, toxic encephalopathy. Hyperpyrexia (≥39℃) accounts for 93.9% (439 cases). The length of fever time ranged from 5 to 17 days and the median fever time was 8.5 days. A total of 16% (78 cases) of the children had an underlying condition (with congenital heart diseases, and surgical diseases the most common condition). The stay of hospital ranged from 1 to 41 days, the median stay of hospital was 5.6 days (3–7 days). 55 children (11.3%) required intensive care, and 2 (0.4%) died (Table 1).

**Table 1:** Clinical features of HAdV-positive respiratory infection in hospitalized children

Variables	Clinical characteristics	Number of HAdV-positive children, n (%)
Clinical manifestation	Fever	456 (93.4)
	Hyperpyrexia (≥ 39 ℃)	439 (90.0)
	Duration of fever between 7 and 10 days	277 (56.8)
	Duration of fever > 10 days	96 (19.7)
	Cough	462 (94.7)
	Wheezing	128 (26.2)
	Somnolence	87 (17.8)
	Shortness of breath	72 (14.8)
	Pertussoid	9 (1.8)
	Vomiting and diarrhea	37 (7.6)
	Convulsion and altered mental status	31 (6.4)
	Bulbar conjunctival congestion	13 (2.7)
	Urinary irritation symptoms	4 (0.8)
Laboratory test	High WBC count	28 (5.7)
	Decreased WBC count	11 (2.3)
	High CRP (> 8 mg/L)	40 (8.2)
	High PCT (> 0.5 ng/ml)	11 (2.3)
Underlying diseases	Congenital heart diseases	16 (3.3)
	Surgical diseases	13 (2.7)
	Malnutrition	12 (2.5)
	Arrhythmia	10 (2.1)
	Epilepsy	5 (1.0)
	Asthma	3 (0.6)
Sequelae	Bronchiectasia	3 (0.6)
	Bronchiolitis obliterans	3 (0.6)

WBC, white blood count; CRP, C-reaction protein; PCT, procalcitonin; congenital heart disease, including atrial septal defect, ventricular septal defect, patent ductus arteriosus, aortic and tricuspid valve disease; surgical diseases, including crissum abscess, hiatal hernia, indirect inguinal hernia, congenital esophageal atresia, hydrocephalus, hemangioma, Scoliosis, chonechondrosternon, cholecystolithiasis

### Complications of the HAdV-positive inpatients

Of the 488 HAdV-positive children with ATIs, 40 cases presented acute respiratory insufficiency, of which, 21 cases with acute respiratory failure. 138 cases occurred extrapulmonary symptoms (including hepatic injury (6.4%, 31/488); marrow suppression (5.3%, 26/488); myocardial injury (3.5%, 17/488) and 2 cases with laryngemphraxis, cerebral infarction, and coagulation abnormalities respectively (Table 2). 368 cases had taken imageological examination (such as chest radiography, chest CT scan, and ultrasound). Intrapulmonary complications and intrathoracic complications were displayed in Table 2.

**Table 2:** Complication of HAdV-positive respiratory infection in children

Variables	Complications	Number of HAdV-positive children, n (%)
Intrapulmonary and intrathoracic complications	Pulmonary consolidation	108 (29.3)
	Pulmonary emphysema	86 (23.4)
	Pulmonary atelectasis	56 (15.2)
	Serous effusion	57 (15.4)
	Pleural effusion	56 (15.2)
	Acute respiratory insufficiency	40 (8.2)
	Respiratory failure	21 (4.3)
	Pericardial effusion	8 (2.17)
	Necrotizing pneumonia	1 (0.2)
Extrapulmonary symptoms	Hepatic injury	31 (6.4)
	Marrow suppression	26 (5.3)
	Myocardial injury	17 (3.5)
	Coagulation abnormalities	2 (0.4)
	Cerebral infarction	2 (0.4)
	Laryngemphraxis	2 (0.4)
	Ascitic fluid	2 (0.4)
	Pelvic effusion	2 (0.4)
	Hemophagocytic syndrome	1 (0.2)

### Coinfection with other respiratory viruses

Of 488 inpatients, 275 (56.4%) had HAdV single infection, and 213(43.6%) comprised co-infection with other respiratory pathogens (including 191(89.7%, 191/213) with one other pathogen, 22 (10.3%, 22/213) with two other pathogens). Influenza virus (24.4%, 52/213), parainfluenza virus (18.8%, 40/213), human respiratory syncytial virus (13.6%, 29/213) were the most three frequently identified viruses in co-infected cases. The proportions of fever, hyperpyrexia, duration of fever >10 days, severe pneumonia, and wheezing in the co-infection group were remarkably higher than those in HAdV single-infection group (all *p* < 0.05) and the stay of the hospital in the co-infection group was longer than HAdV single infection group (Z = − 8.805, *p* = 0.000) (Table 3). There was no significant difference between the single-infection group and the co-infection group in every age group (χ^2^=1.497, *p*=0.683).

**Table 3:** Comparison of clinical characteristics of children with HAdV co-infection and single infection [n (%)]

Characteristics	Single-infection (n = 275)	Co-infection (n = 213)	χ^2^/z	*P* value
Age (years)			1.497	0.683
< 0.5	4 (1.5)	2 (0.9)		
≥ 0.5 to 2	73 (26.5)	50 (23.5)		
≥ 2 to 5	152 (55.3)	118 (55.4)		
≥ 5	46 (16.7)	43 (20.2)		
Gender (male)	175 (63.6)	130 (61.0)	0.347	0.556
Duration of hospitalization [M (Q1, Q3)]	4.0 (3.0,5.0)	6 (4.0,9.0)	− 8.805	0.000
Fever	247 (89.8)	209 (98.1)	13.508	0.000
Hyperpyrexia (≥ 39 ℃)	236 (85.8)	203 (95.3)	11.960	0.001
Duration of fever > 10d	43 (15.6)	53 (24.9)	6.494	0.011
Wheezing	31 (11.3)	97 (45.5)	72.841	0.000
Underlying diseases	37 (13.5)	41 (19.2)	3.001	0.083
Severe pneumonia	23 (8.4)	63 (29.6)	37.209	0.000
High WBC count	12 (40.0)	16 (30.2)	0.825	0.364
High CRP (> 8 mg/L)	13 (43.3)	27 (50.9)	0.444	0.505

M, median; Q1, first quartile; Q3, third quartile; WBC, white blood count; CRP, C-reaction protein

### Comparison between severe and non-severe pneumonia

The proportions of duration of hospitalization, duration of fever >10 days, wheezing, shortness of breath, change in level of consciousness, serosal fluids, extrapulmonary symptoms, co-infections and underlying diseases were significantly higher in severe pneumonia group than those in the mild pneumonia group (all *p* < 0.05). The proportion of children with severe pneumonia in the ≥ 6 months to < 2 years group was higher than that in the ≥ 2 to < 5 years and ≥ 5 years groups (*p* < 0.05) (Table 4).

**Table 4:** Comparison of clinical characteristics of HAdV-positive children with severe and non-severe pneumonia [n (%)]

Characteristics	Severe cases (n = 86)	Non-severe cases (n = 257)	χ^2^/z	*P* value
Age (years)			10.077	0.018
< 0.5	2 (2.4)	2 (0.8)		
≥ 0.5 to 2	31 (36.0)	54 (21.0)		
≥ 2 to 5	38 (44.2)	154 (59.9)		
≥ 5	15 (17.4)	47 (18.3)		
Gender (male)	47 (54.7)	167 (65.0)	2.930	0.087
Duration of hospitalization [M (Q1,Q3)]	8 (6.0,11.0)	4 (3.0,6.0)	− 9.182	0.000
Fever	86 (100.0)	253 (98.4)	1.354	0.245
Hyperpyrexia (≥ 39℃)	79 (91.9)	250 (97.3)	4.828	0.060
Duration of Fever > 10 days	35 (40.7)	40 (15.6)	23.825	0.000
Wheezing	56 (65.1)	61 (23.7)	49.094	0.000
Shortness of breath	54 (62.8)	11(4.3)	143.627	0.000
Change in level of consciousness	66 (76.7)	4 (1.6)	219.659	0.000
Serosal fluids	39 (45.3)	13 (5.1)	81.327	0.000
Extrapulmonary symptoms	30 (34.9)	51 (19.8)	8.080	0.004
Co-infection	63 (73.3)	114 (44.4)	21.546	0.000
Underlying diseases	24 (27.9)	33 (12.8)	10.556	0.001

### HAdV species and type distribution

In the study period, 162 HAdV-positive samples were randomly selected for hexon gene amplification and Sanger sequencing, and the specimens were subjected to further molecular typing. 155 (95.7%) were successfully confirmed for the HAdV genotypes. They included species B (140, 90.3%), species C (7, 4.5%), species E (6, 3.9%3), species D (2, 1.3%). Species B was the most common genotype, followed by Species C, Species D, and Species E. HAdV-B3 and HAdV-B7 were the most predominant detect genotypes. The distribution of HAdV genotypes of enrolled inpatient with ARIs was shown in Fig. 2B. In addition, HAdV-B3 caused febrile respiratory infection in all age groups. The distribution of HAdV genotypes among different age groups of inpatients with ARIs was shown in Table 5. The HAdV genotype distribution in different severity groups showed that HAdV-B3 occurred commonly among three groups, of which, HAdV-B55 occurred only in the severe pneumonia group (Table 6). Two cases of HADV-B55 died in this study. In our study, HAdV-B7 presented only in patients with pneumonia, of which, the HAdV-B7 detection rate in the severe pneumonia group was significantly higher than the non-severe pneumonia group (χ^2 ^= 95.857, *p *= 0.000).

**Table 5:** HAdV genotype distribution of inpatient with ARIs in different age group [n (%)]

	< 6 months	6 months–2 years	2–5 years	≥ 5 years
HAdV-B3	5 (3.2)	45 (29.0)	24 (15.5)	13 (8.4)
HAdV-B7	NA	30 (19.4)	14 (9.0)	4 (2.6)
HAdV-B55	NA	4 (2.6)	NA	NA
HAdV-B21	NA	1 (0.6)	NA	NA
HAdV-C2	NA	3 (1.9)	2 (1.3)	NA
HAdV-C5	NA	1 (0.6)	1 (0.6)	NA
HAdV-D53	NA	2 (1.3)	NA	NA
HAdV-E4	NA	4 (2.6)	2 (1.3)	NA

NA, not applicable

**Table 6:** Distribution of HAdV genotypes in different severity groups among ARIs inpatients [n (%)]

	Severe pneumonia	Non-severe pneumonia	Non-pneumonia
HAdV-B3	16 (10.3)	60 (38.7)	11 (7.1)
HAdV-B7	33 (21.3)	15 (9.7)	NA
HAdV-B55	4 (2.6)	NA	NA
HAdV-B21	NA	1 (0.6)	NA
HAdV-C2	2 (1.3)	1 (0.6)	2 (1.3)
HAdV-C5	1 (0.6)	1 (0.6)	NA
HAdV-D53	NA	2 (1.3)	NA
HAdV-E4	1 (0.6)	3 (1.9)	2 (1.3)

NA, not applicable

## Discussion

HAdV was known as the most frequent cause of ARIs in children which is a highly contagious pathogen. It is reported that the detection rate of HAdV infection varied in different months, seasons, ages and regions, and circulated all year round, with epidemic outbreaks and clusters of severe cases occurring in individual regions [2, 15, 16]. Some HAdV types are common causes of severe respiratory tract infections in children. The majority of adenovirus respiratory infections are light to moderate and self-limited; however, sometimes they may cause life-threatening conditions, comorbidities, and serious sequelae [17–19]. The prognosis of severe HAdV infection in pediatric patients is poor, and many sequelae may be left, such as, bronchiectasis, interstitial fibrosis, etc., which can be life-threatening in severe cases [2, 20]. Edmond et al. [17] reported that association between adenovirus infection and higher risk (54.8% [39.2–70.5%]) for long-term sequelae compared to other causes of pneumonia in a meta-analysis of pneumonia in children younger than 5 years.

The present study retrospectively analyzed the epidemiology, clinical features, and genotype of 488 hospitalized children with adenovirus positive for acute respiratory tract infection in our hospital from September 2018 to August 2019. The overall detection rate of 6.9% of HAdV in hospitalized children with ARIs during the study period was generally consistent with our and other countries’ detection results [21, 22].Our study showed the peak detection rate of HAdV in children was in aged 6 months and < 2 years and suggesting that young children may be sensitive to HAdV in particular, which was consistent with that reported in multiple centers of China [23]. We noted that more than 80% of HAdV-positive ARIs were between the ages of 6 months and less than 5 years which was consistent with other studies [24, 25]. Due to the protection of maternal antibodies, the detection rate was only 1.2% in HAdV-positive cases less than 6 months. Pereira et al. [26] reported that children over 5 years old may have strong immunity to adenovirus infection. These results were consistent with Guangzhou reports in 2012 [27].

The clinical presentation of HAdV-positive ARIs is associated with age, genotype, and host immune function status. Previous studies have shown that age < 5 years, underlying diseases, immunocompromised status were more likely to be infected and developed severe HAdV-positive infection [28]. We noted that fever and coughwere the most frequent symptoms observed in HAdV-positive hospitalized cases with ARIs, and the majority of children were less than 5 years old which is similar to the Italian study [29]. Our study showed that more than 70% of HAdV-positive hospitalized ARIs were diagnosed with pneumonia, and nearly one quarter of HAdV-positive pneumonia patients progressed to severe disease. Wheezing and shortness of breath was more frequent in the severe group. Patients with severe HAdV infections have prolonged fever and required longer hospitalization which was consistent with Wuhan report [30]. It is different in severity in HAdV infection under the age, environmental status, and immunological status of the patient. Clinician can’t distinguish HAdV-positive ARIs from other pathogens infections (such as Mycoplasma pneumoniae, Influenza, etc.) because the clinical presentation seemed to be alike.

Many studies have reported co-infections with other respiratory pathogens [3, 31]. This study suggests that more than 40% of patients with HAdV-positive ARIs have mixed infections. The co-infection rate of adenovirus infection in Taiwan was 19% from 2002 to 2011 [32], and it was as high as 64% in Guanzhou [27]. Co-infection rates in pediatric patients with HAdV-positive ARIs are associated with geographic regions and different detection methods. When comparing the clinical data of children with co-infection and single with HAdV infection, the proportion of children with fever, fever duration >10 days, severe pneumonia and wheezing and days of hospitalization were higher in the mixed infection group than in the single infection group. Therefore, pediatric clinicians should be alert to the concurrent co-infection of HAdV with other pathogens, leading to aggravation, prolongation of the disease course, and more difficult treatment.

According to previous studies, HAdV- B (3, 7, 21), HAdV-C (1, 2, 5, 6) and HAdV-E4 were the most frequent HAdV species which caused ARIs in children worldwide. HAdV-3 and HAdV-7 were the most frequent genotypes in China [33]. HAdV-7 is closely related to severe pneumonia and high mortality compared with other genotypes in infants and children [34]. Although patients from every age group may be susceptible to HAdV infections, more cases of HAdV infections were observed among less than 4 years old children in this study. In this study, HAdV-B was the most commonly identified genotype compared with other HAdV types, suggesting that young children with ARIs may be particularly susceptible to species B. HAdV-B3was the dominant genotype in cases with HAdV respiratory infection.

A nationwide epidemiological surveillance program for HAdV infection has not yet been built in China currently. HAdV identification is usually conducted for laboratory research and it is hard to be applied to the routine clinical diagnosis. We need long-time surveillance and rapid diagnostic methods of HAdV infection. Rapid detection is important to avoid unnecessary antibiotic consumption.

This research had certain limitations. Firstly, this is a one-year single-center study and these results didn’t generalize to other children across China; Secondly, we were unable to proceed with comparative analyses between genotypes owing to small sample size; thirdly, due to the retrospective analysis, we were unable to record all the symptoms and signs which may cause exposure misclassification.

## Conclusions

Our study showed that HAdV detection rate among respiratory infections is related to age and season and bronchopneumonia accounts for about 70% of HAdV-positive inpatients. The common clinical manifestations include hyperpyrexia, cough, wheezing, and shortness of breath. HAdV-B3 and HAdV-B7 are the most common detected types in children with respiration infections which occur in different severity groups. HAdV-B55 occur only in the severe group.

## Data Availability

Not applicable.

## Abbreviations

HAdV: Human adenovirus
ARIs: Acute respiratory infections
NPAs: Nasopharyngeal aspirates
DFA: Direct immunofluorescence assay

## References

[1] Finianos M, Issa R, Curran MD (2016). Etiology, seasonality, and clinical characterization of viral respiratory infections among hospitalized children in Beirut, Lebanon. J Med Virol.

[2] Sandkovsky U, Vargas L, Florescu DF (2014). Adenovirus: current epidemiology and emerging approaches to prevention and treatment. Curr Infect Dis Rep.

[3] Jain S, Williams DJ, Arnold SR (2015). Community-acquired pneumonia requiring hospitalization among US children. N Engl J Med.

[4] Xie L, Zhang B, Xiao N (2019). Epidemiology of human adenovirus infection in children hospitalized with lower respiratory tract infections in Hunan. China J Med Virol.

[5] Lion T (2014). Adenovirus infections in immunocompetent and immunocompromised patients. Clin Microbiol Rev.

[6] Zhang SY, Luo YP, Huang DD (2016). Fatal pneumonia cases caused by human adenovirus 55 in immunocompetent adults. Infect Dis (Lond).

[7] Tórtora RP, Guimarães M, de Souza LM (2015). Adenovirus species C detection in children under four years of age with acute bronchiolitis or recurrent wheezing. J Clin Virol.

[8] Yu P, Ma C, Nawaz M (2013). Outbreak of acute respiratory disease caused by human adenovirus type 7 in a military training camp in Shaanxi. China Microbiol Immunol.

[9] Lin YC, Lu PL, Lin KH (2015). Molecular epidemiology and phylogenetic analysis of human adenovirus caused an outbreak in Taiwan during 2011. PLoS ONE.

[10] Qin L, Leyun X, Bing Z (2019). Epidemiological investigation of adenovirus pneumonia in children in Hunan Province. Chin Pediatric Emerg Med.

[11] Wen S, Lin Z, Zhang Y (2021). The Epidemiology, molecular, and clinical of human adenoviruses in children hospitalized with acute respiratory infections. Front Microbiol.

[12] Mennechet FJD, Paris O, Ouoba AR (2019). A review of 65 years of human adenovirus seroprevalence. Expert Rev Vaccin.

[13] Bradley JS, Byington CL, Shah SS (2011). The management of community-acquired pneumonia in infants and children older than 3 months of age: clinical practice guidelines by the Pediatric Infectious Diseases Society and the Infectious Diseases Society of America. Clin Infect Dis.

[14] Lu X, Erdman DD (2006). Molecular typing of human adenoviruses by PCR and sequencing of a partial region of the hexon gene. Arch Virol.

[15] Wang W, Liu Y, Zhou Y (2017). Whole-genome analyses of human adenovirus type 55 emerged in Tibet, Sichuan and Yunnan in China, in 2016. PLoS ONE.

[16] Yu Z, Zeng Z, Zhang J (2016). Fatal community-acquired pneumonia in children caused by re-emergent human adenovirus 7d associated with higher severity of illness and fatality rate. Sci Rep.

[17] Edmond K, Scott S, Korczak V (2012). Long term sequelae from childhood pneumonia; systematic review and meta-analysis. PLoS ONE.

[18] Larrañaga C, Kajon A, Villagra E (2000). Adenovirus surveillance on children hospitalized for acute lower respiratory infections in Chile (1988–1996). J Med Virol.

[19] Carballal G, Videla C, Misirlian A (2002). Adenovirus type 7 associated with severe and fatal acute lower respiratory infections in Argentine children. BMC Pediatr.

[20] Lynch JP, Kajon AE (2016). Adenovirus: epidemiology, global spread of novel serotypes, and advances in treatment and prevention. Semin Respir Crit Care Med.

[21] Jin Y, Zhang RF, Xie ZP (2013). Prevalence of adenovirus in children with acute respiratory tract infection in Lanzhou. China Virol J.

[22] Barnadas C, Schmidt DJ, Fischer TK (2018). Molecular epidemiology of human adenovirus infections in Denmark, 2011–2016. J Clin Virol.

[23] Duan YL, Zhu Y, Xu BP (2019). Multicenter study of human adenovirus infection in pediatric community-acquired pneumonia in China. Zhonghua Er Ke Za Zhi.

[24] Kwon HJ, Rhie YJ, Seo WH (2013). Clinical manifestations of respiratory adenoviral infection among hospitalized children in Korea. Pediatr Int.

[25] Wu PQ, Zeng SQ, Yin GQ (2020). Clinical manifestations and risk factors of adenovirus respiratory infection in hospitalized children in Guangzhou, China during the 2011–2014 period. Medicine (Baltimore).

[26] Pereira MS (1973). Adenovirus infections. Postgrad Med J.

[27] Zou L, Zhou J, Li H (2012). Human adenovirus infection in children with acute respiratory tract disease in Guangzhou. China Apmis.

[28] Gray GC, McCarthy T, Lebeck MG (2007). Genotype prevalence and risk factors for severe clinical adenovirus infection, United States 2004–2006. Clin Infect Dis.

[29] Esposito S, Zampiero A, Bianchini S (2016). Epidemiology and clinical characteristics of respiratory infections due to adenovirus in children living in Milan, Italy, during 2013 and 2014. PLoS ONE.

[30] Xu N, Chen P, Wang Y (2020). Evaluation of risk factors for exacerbations in children with adenoviral pneumonia. Biomed Res Int.

[31] Meyers L, Ginocchio CC, Faucett AN (2018). Automated real-time collection of pathogen-specific diagnostic data: syndromic infectious disease epidemiology. JMIR Public Health Surveill.

[32] Lai CY, Lee CJ, Lu CY (2013). Adenovirus serotype 3 and 7 infection with acute respiratory failure in children in Taiwan, 2010–2011. PLoS ONE.

[33] Liu C, Xiao Y, Zhang J (2015). Adenovirus infection in children with acute lower respiratory tract infections in Beijing, China, 2007 to 2012. BMC Infect Dis.

[34] Huang X, Yi Y, Chen X (2021). Clinical characteristics of 204 children with human adenovirus type 7 pneumonia identified by whole genome sequencing in Liuzhou. China Pediatr Infect Dis J.

